# An Overview of Flexible Sensors: Development, Application, and Challenges

**DOI:** 10.3390/s23020817

**Published:** 2023-01-10

**Authors:** Enze Liu, Zhimin Cai, Yawei Ye, Mingyue Zhou, Hui Liao, Ying Yi

**Affiliations:** School of Mechanical Engineering and Electronic Information, China University of Geosciences, Wuhan 430074, China

**Keywords:** flexible sensor, strain sensor, humidity sensor, human machine interaction

## Abstract

The emergence and advancement of flexible electronics have great potential to lead development trends in many fields, such as “smart electronic skin” and wearable electronics. By acting as intermediates to detect a variety of external stimuli or physiological parameters, flexible sensors are regarded as a core component of flexible electronic systems and have been extensively studied. Unlike conventional rigid sensors requiring costly instruments and complicated fabrication processes, flexible sensors can be manufactured by simple procedures with excellent production efficiency, reliable output performance, and superior adaptability to the irregular surface of the surroundings where they are applied. Here, recent studies on flexible sensors for sensing humidity and strain/pressure are outlined, emphasizing their sensory materials, working mechanisms, structures, fabrication methods, and particular applications. Furthermore, a conclusion, including future perspectives and a short overview of the market share in this field, is given for further advancing this field of research.

## 1. Introduction

Recent progress in electronic systems has ignited promising applications in many fields, including consumer electronics [1,2], human–computer interactions [3,4], augmented reality devices [5,6], and electronic skins [7,8]. A sensor that can sense the physical world is an essential part of these systems. Conventional sensors are generally fabricated from semiconductors that have rigid substrates or scaffolds that become deformable once they are thinned and oriented into nanostructures [9], thus limiting their applications such as wearable “smart” devices [10], soft robots [11,12], body motion tracking [13,14], and portable medical diagnostic devices [15,16]. The emergence of flexible sensors made of inherently elastic materials, such as hydrogels and organic semiconductors, may allow the revolutionary development of electronic systems because of their significant advantage of allowing a high degree of design freedom. A flexible sensor can be folded into different shapes and even trimmed down to different sizes, thus greatly enlarging its fields of application. For example, the integration of wearable electronics could meet the softness demands of clothing or fit the irregular surfaces of tissues and the body.

The manufacture of flexible sensors needs novel designs and appropriate materials, which mainly include conductors and synthesized materials. Conductors are generally classified into the carbon family and the metal oxides (and sulfides). For example, graphene is one of the most popular two-dimensional (2D) nanostructure-based semiconductors in the carbon family [17]. Non-transition-metal oxides such, as ZnO and SnO_2_, which exhibit high sensitivity and favorable conductivity, are also widely used as sensing materials [17]. As a representative synthesized material, the emergence of MXene (e.g., Ti_3_C_2_Tx) has greatly widened the selection of materials that are suitable for flexible sensors because of its unique sandwich-like layer structure, its excellent electrical conductivity, and its large area of hydrophilicity [18]. Furthermore, organic semiconductors with features such as π-conjugation, low cost, print compatibility, solution processability, and light weight are other common sensing materials used for flexible sensors [19].

Alongside the materials, the substrate or structure is another core factor that affects the particular demands and application of a sensor [20,21,22]. For example, to apply a flexible sensor in wearable electronics, the designer must select a proper substrate material and design that can withstand high strain values. Moreover, the substrate needs excellent ability to integrate with the sensing element without affecting the device’s reliability and digital signal readout [23]. The popular materials that have been adopted as flexible substrates include polyimide (PI) [24], polyetheretherketone [25], polyethersulfone (PES) [26], polycarbonate, poly(ethylene naphthalene) (PEN), hydrogel [27], and polyethylene terephthalate (PET) [28]; however, their thermal stability, light transmittance, electrical conductivity, chemical stability, and mechanical elasticity should be considered prior to their practical application.

In this work, we selected noteworthy studies that aimed to demonstrate different types of flexible sensors, rather than presenting all the relevant reported works in the literature. For clarity, the most recent state-of-the-art flexible humidity sensors and flexible strain/pressure sensors were adopted as examples to suggest the key future trends and developments of flexible sensors. The following subsections present the selection of materials, structures, working mechanisms, and specific applications of flexible sensors for sensing humidity and strain/pressure, and summarize the fabrication methods of the corresponding sensors. Finally, a conclusion is provided, and the challenges of these emerging devices are addressed for inducing the smooth exchange of new ideas and research interests.

## 2. Flexible Sensors for Sensing Humidity

Humidity sensors that can sense the presence or amount of water vapor in the atmosphere or other environments have been widely applied in food monitoring, environmental monitoring, human–computer interactions, etc. [29]. An ideal humidity sensor requires high sensitivity, fast responses, a short recovery time, a wide monitoring range, excellent durability, low cost, and high reproducibility [30]. In this subsection, the sensing mechanisms, materials, and applications of humidity sensors are presented.

### 2.1. Sensing Mechanisms

**Figure 1:**
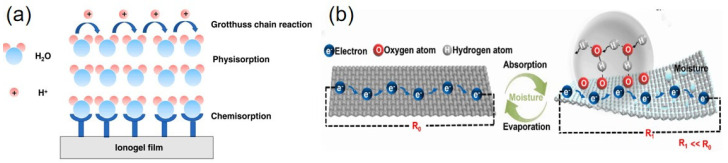
Example of the Grotthuss mechanism: (**a**) illustration of the humidity-sensing mechanism of the PIL (reproduced with permission from [32]); (**b**) principle of the OCF sensor (reproduced with permission from [33]).

The processes of sensing humidity typically involve changes in either the conductivity or the capacitance of the sensing material. In 1806, Grotthuss observed the decomposition process of water and the occurrence of some type of “action” that was transferred along the line or chain of molecule formation [31]. The contemporary proton jumping theory is based on the Grotthuss mechanism. This indicates that the transfer of protons occurs through a series of hydrogen bonds between hydrated hydrogen ions and water molecules, as shown in Figure 1a. The adsorption of water molecules can be divided into two processes: physical adsorption and chemical adsorption. First, the water molecules occupy the water affinity point through chemical adsorption. Next, the water molecules are adsorbed on the chemically adsorbed molecular layer of water through a single hydrogen bond, and the free protons jump through the physically adsorbed water molecules, thus increasing the conductivity and capacitance based on the hopping process of the proton, which can be expressed as [32]

$$H_{2}O+H_{3}O^{+}\to H_{3}O^{+}+H_{2}O$$

Yi et al. generated carbonized fabric (CF) through the high-temperature carbonization of cellulose and then immersed the CF in an acidic mixture to promote the growth of oxygen-containing functional groups on the surface of the CF. The oxygen-containing functional groups provided sufficient adsorption sites for the water molecules. Continuous water molecules connected by hydrogen bonds underwent proton jumps, causing changes in the resistance of the oxidated carbonized fabric (OCF), which could reflect the corresponding changes in humidity (Figure 1b) [33].

In addition to the structures with the capacity for “proton jumps”, those with dielectric layers in between the electrodes usually act as capacitance-based humidity sensors. Generally, the formula used to link the capacitance output of the sensor and the varying dielectric constant of the sensing material in different wet environments is expressed as follows [34]:

(1) $$C_{p}=\frac{\varepsilon_{r}\varepsilon_{0}}{d}S$$

where *C_p_* is the sensor’s capacitance, *ε*_0_ is the dielectric constant of the sensing material, *ε_r_* is the dielectric constant in air, *S* is the squared area of the capacitor poles, and *d* is the distance between the capacitor poles. At a given temperature, *ε*_0_ is a definite value. *S* and *d* are also definite values when the shape and size of the device have been determined. The sensing material absorbs polar water molecules, resulting in enhanced polarization and increased *ε_r_*, which, in turn, increase the output, *C_p_*. Zhang et al. prepared a new printed parallel plate electrode-based humidity sensor that enabled contact with water vapor [35], and it had a capacitance of up to several hundred pF higher than that provided by the typical forked finger electrode because of the larger front-to-back area of the capacitance poles’ plates.

Resistance-based humidity sensors are usually simple in their structural design and have the advantages of less cost, low power consumption, and simple measurement schemes [36]. The sensing mechanisms of resistive humidity sensors are related to the change in conductivity caused by the absorption and resolution of water molecules on the device’s surface. He et al. reported a humidity sensor made from a LiCl salt solution-modified single-walled carbon nanotube (SWNT)/polyvinyl alcohol (PVA) filament, in which the conductivity mainly came from the diffusion and movement of ions [37]. In this case, Li^+^ and Cl^−^ were generated during the dissolution of LiCl in water, thus dominating the carrier transport-based mechanism. As the humidity level increased, more Li^+^ and Cl^−^ were generated by the deliquescence of LiCl; accordingly, the ion concentration increased, resulting in increased carrier mobility, which decreased the material’s impedance.

Surface acoustic wave (SAW)-based mechanisms have also been widely adopted to design humidity sensors [38]. The transmission speed or frequency of the SAW changes when water molecules contact the surface of a SAW-based humidity sensor. Accordingly, the change in frequency can be expressed as follows [39]:

(2) $$\Delta f=\frac{Cf_{r}^{2}}{A}\Delta m$$

where *f_r_* represents the resonant frequency, *C* is a constant, Δ*m* is the mass change caused by the absorption of water, and *A* denotes the sensing area. Although SAW humidity sensors have the advantages of high sensitivity, a miniaturized structure, stable frequency, and low power consumption [40], they are typically fabricated on rigid substrates [41], showing poor compatibility with flexible electronics.

### 2.2. Materials and Design

The design and sensing materials are the core components of humidity sensors. From the perspective of moisture-sensitive materials, materials with porous, pleated, and uniformly stacked structures often exhibit hydrophilic characteristics [34]. In addition to their structure, moisture-sensitive materials easily bind with water molecules because of their rich hydrophilic groups [42]. The popular materials used to fabricate the humidity sensor typically involve the carbon family [43], metal oxide (and sulfide) types [44,45], or polymers [46]. We do not intend to list all the relevant work in this review. For clarity, we compare recently reported flexible humidity sensors using the aforementioned material types and the related production methods (Table 1).

**Figure 2:**
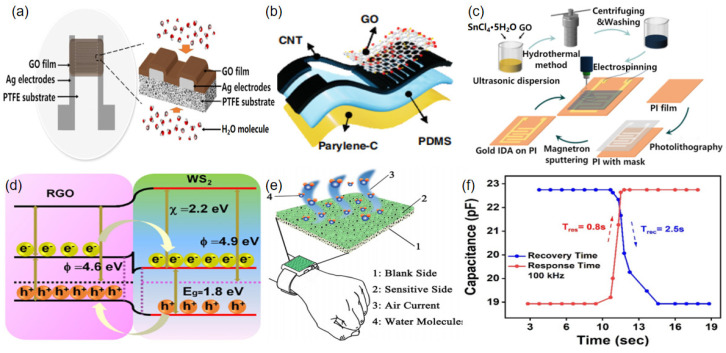
(**a**) Structure of a flexible humidity sensor using graphene oxide and PTFE (reproduced with permission from [47]); (**b**) exploded view of a CNT-based flexible sensor (reproduced with permission from [49]); (**c**) illustration of the process of preparing a SnO_2_/RGO humidity sensor (reproduced with permission from [50]); (**d**) illustration of the different mechanisms of interaction between RGO and WS_2_ (reproduced with permission from [53]); (**e**) illustration of a PANI/PVDF IFHS sensor (reproduced with permission from [55]); (**f**) response and recovery time of a capacitive humidity sensor at 10 kHz (reproduced with permission from [56]).

The members of the carbon family are the most popular materials for fabricating humidity sensors, e.g., graphene oxide (GO) and its derivatives. Wei et al. prepared a capacitive flexible humidity sensor using ethanol-treated hydrophobic porous polytetrafluoroethylene (PTFE) as a substrate and graphene oxide (GO) as a moisture-sensitive material (Figure 2a) [47]. The PTFE substrate greatly enhanced the amount of water molecules adsorbed, due to the porous structure and the excellent hydrophilicity of ethanol. Li et al. developed a crinkled graphene oxide (CGO) film-based sensor by pre-stretching shape-memory polylactic acid fibers (SMPLAF) [48]. The unique crinkled morphology of the CGO films provided a large specific surface area and a high capillary force, which facilitated the absorption of water molecules. The as-prepared sensors had an excellent moisture-sensitive humidity range and a stable baseline, which was achieved by adjusting the wavelength of the folded structure. Wang et al. developed a capacitive humidity sensor that consisted of vertically aligned carbon nanotube (VACNT) electrodes, a PDMS/parylene C double-layer substrate, and GO, which acted as a moisture-sensitive material (Figure 2b) [49]. Although the device had a simple structure, it displayed an ultra-fast response (20.8 ms) and recovery time (19.9 ms), high sensitivity (16.7 pF/% RH), low hysteresis (<0.44%), and high repeatability (2.7%).

As a conventional sensing material, metallic oxides or sulfides also exhibit great potential for fabricating humidity sensors. Yan et al. fabricated a SnO_2_/reduced graphene oxide (RGO) nanocomposite with different reference ratios by the hydrothermal method. Sensitive SnO_2_/RGO films were sprayed on polyimide (PI) films by electrospinning (Figure 2c), and the SnO_2_ nanoparticles were uniformly distributed on the RGO nanosheets [50]. The experimental results revealed that 1 wt% of SnO_2_/RGO had the highest sensitivity to water molecules, while 2 wt% yielded the humidity sensor with the best performance in terms of response/recovery and hysteresis. Farahani et al. prepared a flexible self-powered humidity sensor based on TiO_2_ nanoarrays [51]. The high specific surface area, the nanoporous structure, and the adsorption of water molecules at the Ti^3+^ defect sites made the TiO_2_ nanotube arrays very suitable for sensing humidity. Jin et al. mixed polyvinylpyrrolidone (PVP) with MoS_2_ to fabricate a humidity sensor [52]. The exfoliated MoS_2_ had a nanoscale size, an increased surface-to-volume ratio, and more hydrophilic active sites, thus enhancing the moisture-sensitive properties of the sensor. Zhang et al. used RGO and tungsten disulfide (WS_2_) heterojunctions to design a humidity sensor. The mechanism was based on the transfer of electrons from materials with a low to high work function via the P-N junction formed by RGO and WS_2_, while blocking the electron transfer channels through the potential barriers formed between the P-N heterojunctions. Therefore, the sensitivity of the sensor was improved because the electrons exposed to the outside of the material could adsorb more and more water molecules (Figure 2d) [53].

Polymers are materials that are commonly used to design flexible electronics because of their superior elasticity, excellent mechanical properties, biocompatibility, simple manufacturing process, and flexible mechanical structure [57]. The sensing performance of polymer-based flexible humidity sensors depends mainly on the hydrogen bonds formed between the hydrophilic functional groups of the polymer and the water molecules [58]. Choi et al. prepared sulfonated polyether ether ketone (SPEEK)-based electrospun nanofibers with different degrees of sulfonation and deposited them directly on flexible substrates to fabricate flexible humidity sensors [54]. The results revealed that the higher the degree of sulfonation obtained, the more sulfonyl groups were contained in the PEEK membranes and the greater the electrical conductivity exhibited. Zhao et al. designed flexible humidity sensors (IFHS) by depositing polyaniline (PANI) on poly(vinylidene fluoride) (PVDF) microporous membranes in the presence of a cetyltrimethylammonium bromide (CTAB) surfactant [55]. The PANI/PVDF membranes have a prominent micro/nanostructure, and the increased specific surface area enabled the humidity sensor to exhibit a promising humidity-sensing ability. Moreover, the unilateral deposition of PANI and the high permeability of the integrated flexible humidity sensor (IFHS) avoided direct contact between PANI and human skin, thus mitigating the health concerns (Figure 2e). Khan et al. proposed a humidity sensor using a P(VDF-TrFE)/graphene flower composite as a sensing material, which had a wide humidity monitoring range and a fast response and recovery time (Figure 2f) [56].

### 2.3. Practical Applications

**Figure 3:**
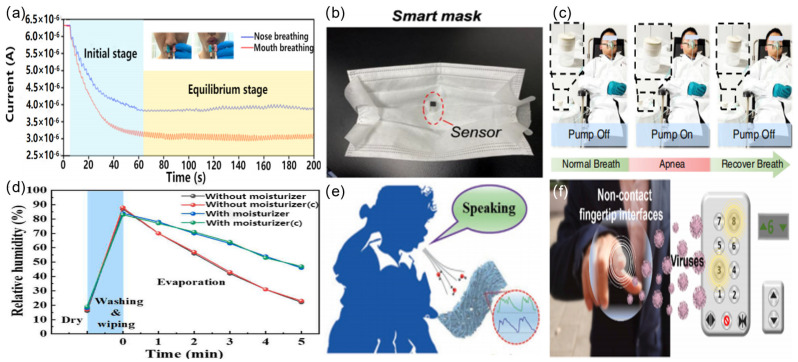
Applications of flexible humidity sensors: (**a**) the current signal profiles of human nose breathing and mouth breathing (reproduced with permission from [65]); (**b**) image of the application of the humidity sensor for monitoring human breath (reproduced with permission from [66]); (**c**) photos of a SAS diagnosing–treating system with an integrated CEH sensor (reproduced with permission from [67]); (**d**) a TiO_2_/CNC humidity sensor used in a moisturization experiment, in which a commercial skin moisture detector was used as a reference (marked as “C”) (reproduced with permission from [68]); (**e**) schematic illustration of MPHS for detecting exhaled air during speaking (reproduced with permission from [69]); (**f**) proof-of-concept of non-contact fingertip interfaces (reproduced with permission from [33]).

Flexible humidity sensors have critical applications in food storage [59], health monitoring [60], contactless sensing [61], human–computer interactions [62], and industrial and agricultural production [63]. As an example of their use in healthcare, humidity sensors can monitor human respiration, which provides critical health information for diagnosing diseases associated with heart attacks [64], asthma, anxiety, and epilepsy. Zhu et al. developed an electrostatic self-assembled paper-based flexible humidity sensor using 2,2,6,6-tetramethylpiperidine-1-oxyl (TEMPO)-oxidized cellulose fibers/carbon nanotube (TOCFs/CNT) composite with different mixture ratios [65]. TC05 was a humidity sensor with a TOCF-to-CNT ratio of 30:1, which was first applied for monitoring oral and nasal respiration separately, as shown in Figure 3a. The change in the response of the curve of mouth breathing is more pronounced, indicating that more moisture is exhaled by the mouth than the nose.

A printed flexible humidity sensor made of a cellulose nanofiber/carbon black (CNF/CB) composite was deployed on a mask to monitor breathing within a relatively narrow space (Figure 3b) [66]. The frequency of breathing could be analyzed through the change in the sensor’s resistance profile. Li et al. prepared a self-powered (CEH) humidity sensor using a graphene oxide (GO)/silk fibroin (SF)/LiBr electrolyte gel in which the power came from the metal–air redox reaction [67]. The sensor was integrated with signal transmission and processing module functions as an integrated respiratory monitoring and diagnostic treatment system, and it could be used for the treatment of sleep apnea (Figure 3c). Wu et al. used TiO_2_/nanocellulose to fabricate a humidity sensor for monitoring changes in moisture in the skin of the arm. The humidity surrounding the arm increases significantly after wiping and washing; the humidity decreased more slowly on the arm with moisturizer compared with the one without moisturizer, and it stabilized at 47% RH after a slow decrease. The results of the as-fabricated sensor precisely matched that of the commercial humidity detector (Figure 3d) [68]. 

Lu et al. made a flexible humidity sensor for speech recognition by anchoring multilayered graphene (MG) into electrospun polyamide (PA) 66 [69]. By detecting the water molecules in the air exhaled by a person during speech, the characteristic peaks of different intensities corresponding to different syllables could be detected, thus enabling speech recognition (Figure 3e). Gong et al. developed a cerium oxide/graphite carbon nitride (CeO2/g-C_3_N_4_) nanocomposite-based self-powered humidity sensor [70]. The sensor exhibits the ability to prevent the diseases associated with sedentary activity. Specifically, the CeO_2_/g-C_3_N_4_ humidity sensor was mounted on a cushion that monitored changes in humidity caused by sedentary behavior and gave a warning when the humidity was high. Furthermore, the humidity sensor also showed potential for application in a human–computer interface. Yi et al. fabricated a freestanding humidity sensor from oxidated carbonized fabric (OCF) and integrated it into control interfaces, such as elevator buttons and combination locks [33]. The sensor allowed people to use fingertip humidity to transmit commands and perform operations (Figure 3f).

## 3. Flexible Strain/Pressure Sensors

The detection of pressure and strain is the first step in perceiving the physical world. Recently, flexible sensors with the capability to stably sense pressure, strain, and even arbitrary deformation have garnered immense interest in various fields, such as healthcare monitoring [71], electronic skin [8], medical diagnosis [72], and human–machine interfaces [73]. The versatility and demands of advanced flexible strain/pressure sensor technology have necessitated their further investigation and development. A brief summary of recent studies on strain/pressure sensors, including their structures and physical characteristics, is provided in Table 2.

### 3.1. Operating Mechanisms

To detect external pressure (or strain) stimuli and then convert them into electrical signals, diverse transduction principles have been extensively explored, including, but not limited to, piezoresistivity [74], capacitance [75], piezoelectricity [76], and triboelectricity [77]. The aforementioned mechanisms have inspired a variety of investigations into stretchable and flexible strain sensors, and they have enriched the physical foundations of this field of research.

The piezoresistive effect generally involves the change in the resistance of the material under externally applied pressure or strain [78]. Generally speaking, the resistance of material can be expressed by the following equation:

(3) $$R=\frac{\rho L}{A}$$

where *ρ*, *L* and *A* represent the resistivity, length, and cross-sectional area, respectively. Owing to their simplicity of fabrication, low cost, and easy signal readout, piezoresistive pressure sensors have become the most widely used [79]. However, there are certain drawbacks hindering such sensors from wider applications. For example, in an effort to achieve stretchability, the sensors are mostly fabricated together with flexible substrates or materials, which may lead to a slower response because of the intrinsic viscoelasticity [80]. Moreover, potential variations in the surrounding temperature could also weaken the practical performance because of the lack of thermal stability [81].

**Table 2:** Summary of the performance of some flexible pressure/strain sensors.

Research Field	Innovation	Sensitivity/GF	Sensing Range	Response Time	Cyclic Stability	Ref.
Conductive materials	Graphene foam	1.16 kPa^−1^	\	150 ms	>10^5^	[82]
	Urchin-like hollow carbon sphere	>10 kPa^−1^	1 Pa–10 kPa	60 ms	>5000	[83]
	Graphene/(CNT) hierarchical networks	GF 197 at 10% strain	50% strain	\	>1000	[84]
	Au film and polyaniline Nanofibers	2.0 kPa^−1^	<3.5 kPa	50 ms	>10^4^	[85]
Structural engineering	RGD spinosum	507 kPa^−1^	0–40 kPa	60 ms	>5000	[86]
	Multiscale and hierarchical wrinkles	GF 1078.1	650% strain	\	>3000	[87]
	Micropyramid arrays	19 kPa^−1^	0.05 Pa–80 kPa	48 ms	>1000	[88]
	Convex microarrays	30.2 kPa^−1^	0.7 Pa–10 kPa	25 ms	>10^5^	[89]
	Graded intrafillable architecture	>220 kPa^−1^	0.08 Pa–360 kPa	9 ms	>5000	[90]

Capacitive pressure sensors are generally composed of two parallel electrodes stacked on two sides of a dielectric layer [91]. The output of a capacitive pressure sensor is the variation in the capacitance caused by the external compression/tension stimuli. Capacitive flexible pressure sensors have a simple structure, low power consumption, and high reliability [92]. The piezoelectric effect generally refers to the generation of electrical potential as a result of electric polarization when mechanical stress is applied to anisotropic crystalline materials [93]. This characteristic of certain dielectric materials endows the sensors with outstanding sensitivity and efficiency [94], enabling effective strain/pressure sensing. However, such sensors cannot have an equal response to static stress, as the polarization of internal charges emerges as an instant dynamic process [95].

The triboelectric effect has been widely reported [96]. Charges are transferred and become concentrated when friction occurs between materials with different electrical properties, thus leading to a detectable electric signal [97]. In the context of pressure and strain sensing, the triboelectric effect has been adopted as a promising mechanism for nanogenerator-based self-powered devices [98]. Therefore, the triboelectric effect can be regarded as a significant energy source instead of as a negative phenomenon, as it was previously regarded [99]. Wang et al. pioneered the development of a flexible triboelectric generator [100] and had a crucial influence on the following related works [101]. It is noteworthy that development of and research into these kinds of sensors are inseparable from the discovery and use of triboelectric materials, and their lifetime is greatly affected by the effects of friction [102]; therefore, further improvements and research are still imperative for their practical utilization.

### 3.2. Materials and Structure

The sensing materials and structures significantly influence the performance of strain/pressure sensors. The selection of appropriate materials must be thoroughly considered prior to designing the sensor. In this subsection, the sensing materials, including the nanofiller type, conductive polymers, and the newly emerged 2D materials, are reviewed.

**Figure 4:**
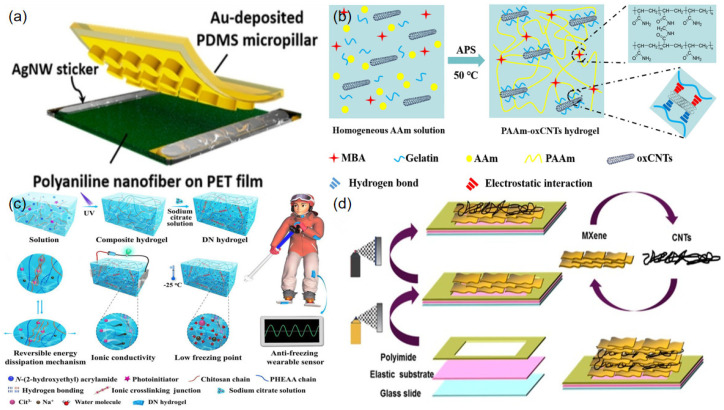
Schematic diagrams of flexible strain/pressure sensors using various materials: (**a**) polyaniline nanofibers and the conductive layer of Au (reproduced with permission from [85]); (**b**) carbon nanotube–reinforced hydrogel (reproduced with permission from [107]); (**c**) CS–PHEAA DN hydrogel (reproduced with permission from [108]); (**d**) Ti_3_C_2_Tx MXene/carbon nanotube composite (reproduced with permission from [110]).

Carbonaceous material and metals are commonly adopted as nanofillers integrated with a flexible substrate to act as strain/pressure sensors. For example, carbon nanotubes (CNTs) and graphene nanoplatelets (GNPs) are two representative materials that have attracted extensive attention because of their outstanding properties and various forms [103]. Huang et al. demonstrated a wearable strain sensor based on multiwalled carbon nanotubes (MWCNT), which were encapsulated by PDMS to prevent the fragile conductive filler from stress-induced damage [104]. The sensor exhibited a rapid response of about 20 ms and outstanding stretchability of 73.2% when applied on a complicated structural surface and human skin. Zhong et al. developed a pressure sensor using wrinkled graphene foams as the sensing material, which exhibited excellent mechanical performance, such as a response time of 150 ms and good stability after 105 loading–unloading cycles [82]. Zinc chloride was introduced when preparing graphene oxide for the purpose of tuning the foam structure. The interlayer distance decreased when withstanding compression stimuli; as a result, the contact area of conductive materials increased, thus leading to a decrease in resistance. Li et al. developed a hierarchical conductive network by combining CNTs and GNPs [84]. When a strain was applied, the CNTs acted as bridges between the separating GNPs, optimizing the electrical connection between the conductive layers, as well as enhancing the stretchability of the sensor without compromising its sensitivity. Park et al. developed a stretchable, highly sensitive pressure sensor array that showed promise in the field of artificial electronic skin and wearable devices [85]. The sensor was fabricated from a microstructure with PDMS micropillars deposited on Au film and a conductive polyaniline nanofiber-coated polyethylene terephthalate (PET) substrate (Figure 4a). Pressure caused a variation in the effective conductive path between the two parts of the sensor, resulting in a change in resistance. The device exhibited a sensitivity of 2.0 kPa^−1^ in the pressure range below 0.22 kPa and a fast response time (50 ms).

In addition to the elastomers, conductive hydrogel are three-dimensional hydrophilic cross-linked networks of natural or synthesized polymers that can also be used as conductive materials [105]. Hydrogels are soft, stretchable, and biocompatible, showing a similar biological nature to tissues [106]. Sun et al. demonstrated a hydrogel for which the performance is enhanced by the integration of oxidized multiwalled carbon nanotubes (oxCNTs) (Figure 4b) [107], while Yang et al. developed a chitosan-poly (hydroxyethyl acrylamide) (CS-PHEAA) double-network hydrogel [108]. The doped free ions contributed to ionic conductivity, while the immobile ones improved the mechanical performance of the hydrogel (Figure 4c). By virtue of a rigid CS ionic network and a soft PHEAA hydrogen network, the as-fabricated hydrogel strain sensor achieved outstanding compressibility (ε = 98%) and superior low-temperature tolerance (as low as −50 °C), which were shown in a frostbite experiment on rats.

MXenes, a family of 2D transition metal carbides, carbonitrides, and nitrides, are newly emerging representative materials with a hydrophilic surface, as well as adjustable electrical, chemical, and mechanical properties [109]. The accordion-like multilayered structure of these materials endows them with variable interlayer distances and inter-atomic distances. As a result, they can exhibit extraordinary conductivity and sensitivity when external pressure or strain is applied. Cai et al. designed an ultra-thin stretchable pressure sensor that was fabricated from composites of Ti_3_C_2_Tx-type MXene and CNTs (Figure 4d) [110]. The overlapping area of the neighboring Ti_3_C_2_Tx layer and the interconnecting conductive pathways of the CNTs changed under pressure. This sensor exhibited an ultra-high sensitivity (a gauge factor of up to 772.6) and excellent mechanical reliability (>5000 cycles), indicating the superiority of MXenes.

**Figure 5:**
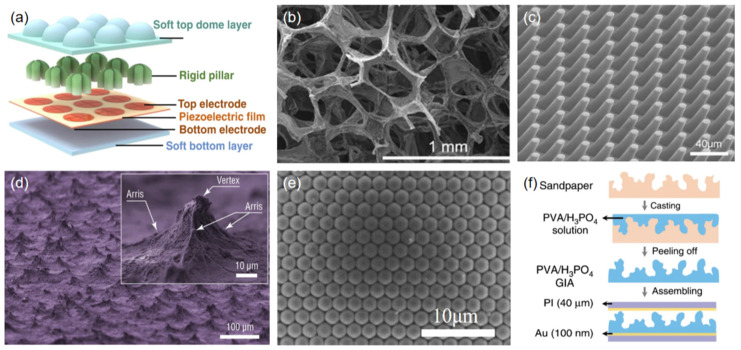
(**a**) Schematic diagram of a finger-shaped sensor (reproduced with permission from [111]); scanning electron microscope (SEM) images of (**b**) porous RGO foam (reproduced with permission from [115]); (**c**) micropillars (reproduced with permission from [116]); (**d**) micropyramids (reproduced with permission from [88]); (**e**) microconvex arrays (reproduced with permission from [89]); (**f**) schematic diagram of the architecture of GIA (reproduced with permission from [90]).

Optimizing the structure is another feasible method for improving sensors’ performance. These structures are usually designed to be bionic or artificially manufactured. The so-called “bionic type” indicates a structure that has a similar biological shape or imitates some biological characteristics. Zhang et al. developed a finger-shaped piezoelectric tactile sensor with exceptional sensitivity (346.5 pC N^−1^ at 30 Hz), overcoming the defects of traditional piezoelectric pressure sensors (Figure 5a) [111]. Owing to their slit organs in the shape of cracks, spiders can sense extremely tiny strains on their cobwebs. Inspired by this mechanism, Kang et al. first reported a nanoscale cracks-based sensor in 2014 [112]. Amjadi et al. developed a controllable parallel cracks-based graphite sensor with a large gauge factor of up to 522.6 [113]. The porous structure is also popular in sensor designs. This was originally inspired by sponges, which have excellent stretchability and a wide detection range. Yao et al. developed a new type of cracks-based microstructured pressure sensor by coating a thin film of graphene oxide nanosheets onto a commercial polyurethane (PU) sponge, which was then immersed into a hot hydrogen iodide solution to obtain rGO as a conductive layer [114]. The as-fabricated flexible pressure sensor showed an outstanding sensitivity of 0.26 kPa^−1^ within a low-pressure range (<2 kPa) and had a minimum detection limit of 9 Pa. Similarly, Golezar et al. fabricated a piezoresistive pressure sensor array based on reduced graphene oxide coated on a PU sponge [115], which had an excellent working range of up to 30 kPa and was capable of providing a pressure distribution map.

The artificially manufactured structures usually involve, but are not limited to, pillars (Figure 5b) [116], pyramids (Figure 5c) [88], and convex arrays (Figure 5d) [117]. Xiong et al. designed a type of convex microarray based on polystyrene (PS) microspheres [89]. This flexible capacitive pressure sensor consisted of Au film electrodes and an ultra-thin PVDF dielectric layer, and demonstrated a super-high sensitivity of 30.2 kPa^−1^ for a tiny pressure range of less than 130 Pa. Yunsik et al. demonstrated a microscale wave-structured capacitive sensor using Ag nanowires as the sensing material (Figure 5e) [118]. This sensor had very high sensitivity (>3.8 kPa^−1^) and a fast response time (<150 ms). Zhou et al. developed a micro/nanoscale hierarchical wrinkle-structured strain sensor [87]. The wrinkles were generated on the surface of Ecoflex as a result of respective ethanol-assisted solidification and soaking in petroleum ether. It is worth mentioning that the volatilization of ethanol during solidification could induce microscale wrinkles, while the nanoscale ones occurred during the process of Ecoflex shrinking. The as-fabricated sensor could stretch to a strain of up to 650% and withstand 3000 cycles under a strain of 200%, and had an ultra-high gauge factor of 1078.1. Bai et al. demonstrated a flexible capacitive pressure sensor based on a graded intra-fillable architecture (GIA) by pouring a mixture of PVA/H_3_PO_4_ onto commercial sandpaper and peeling it off from the surface of the sandpaper after the curing process (Figure 5f) [90]. The contact area was greatly enlarged by the use of this structure, thus contributing to the sensor’s ultra-high sensitivity (S_min_ > 220 kPa^−1^) and extremely high resolution (18 Pa).

### 3.3. Practical Applications

Flexible strain/pressure sensors have various fields of application [119]. We did not intend to review all the relevant works in this subsection. For clarity, we emphasized the practical (or potential) applications of the sensors in the fields of human–machine interactions (HMI) and healthcare monitoring.

**Figure 6:**
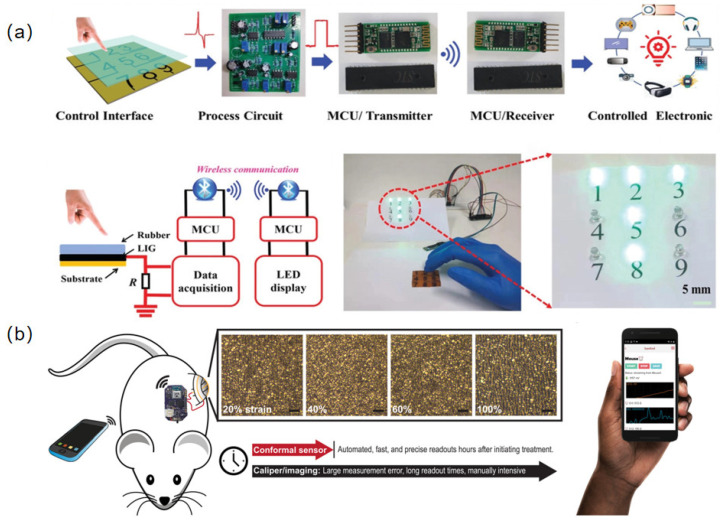
(**a**) Schematic illustration of a wireless control system for real-time motion tracking (reproduced with permission from [125]); (**b**) illustration of a strain sensor and its deployment in a rat model for monitoring the regression of tumors (reproduced with permission from [131]).

As a crucial technology of HMI, flexible pressure sensors are imperative for enhancing artificial intelligence and HMI [120]. HMI can be regarded as a bridge between humans and machines, allowing electronic devices to operate effectively [121]. Flexible sensors are indispensable detection elements of wearable HMI systems, owing to their strong ability to collect information [122]. An example is a flexible sensor that behaves in a similar way to electronic skin, enabling robots to sense their surroundings and spatially detect external stimuli [123]. Equipped with proper circuits, flexible pressure sensors (FPSs) integrated into “smart” gloves can imitate human motion and perform remote tasks [124]. Yan et al. used a new technology, laser direct writing (LDW), to design a flexible high-resolution triboelectric sensor array (TSA), which displayed real-time motion tracking ability [125]. The self-powered version could allow an HMI system to wirelessly control personal electronics (Figure 6a).

In the field of healthcare monitoring, sensors are capable of detecting both small and large changes in compression/tension because of their high gauge factor and stretchability. For example, when deployed onto skin, a variety of physiological activities, including vibration of the throat muscles [126], the bending of fingers [77] and knees [127], the wrist pulse [128], and even vigorous sports [129], can be monitored through the application of these sensor. As a supplementary method in the field of medicine, FPSs are promising candidates for monitoring a patient’s breath, pulse, and heart rates or other physiological indices. Boutry et al. developed a flexible pressure sensor using biodegradable materials for the wireless monitoring of blood flow [130]. Unlike previously reported implantable devices, this sensor disappears after an extended usage period, thus reducing the risk of trauma. The key technology has been demonstrated in experiments on rats. In addition, Alex et al. developed an electronic strain sensor for estimating the size of tumors (Figure 6b) [131]. Compared with the traditional methods of diagnosis, such as bioluminescence and CT, that may have a risk of toxicity, economic issues, or concern about radioactivity, the application of such sensors, which was clinically validated in a rat experiment, paves the way toward new diagnostic technology without creating the aforementioned problems.

## 4. Perspectives and Conclusions

Considering that most electronics and sensors must be applied in scenarios where arbitrarily curved interfaces exist, the mechanical properties of a sensor (specifically, the flexibility) are essential for improving its adaptability to fit the irregular surface. Furthermore, flexible sensors have a wider selection of materials, much easier fabrication procedures, and much lower manufacturing costs than conventional rigid-substrate sensors, thus holding significant promise for extensive innovative applications in various fields, including disease prevention, healthcare monitoring, and artificial electronic skin. This point can be illustrated by the market share of flexible sensors and related products. IDTechEx reported that the markets for wearable and printed sensors are expected to reach USD 5.5 billion and USD 8 billion by 2025 [17], while the market for flexible sensors is expected to be larger than that of wearable and printed sensors, because the former have wider areas of application.

Flexible humidity sensors are regarded as the fastest-growing sensor types, and flexible strain/pressure sensors are among the most sophisticated sensor types [17]. These two types were selected as representative examples to demonstrate the rapid advances in the sensing materials, structures, and processing approaches. However, some challenges remain for the development of more advanced flexible sensors in future research. First, for flexible sensors that are deployed on human skin or other body organs, the biocompatibility and appropriateness of the materials need to be thoroughly analyzed; for example, long-term usage of gas-impermeable or toxic materials may cause irritation and inflammation of the skin [132]. Second, improvements in both the mechanical performance (e.g., elasticity and robustness) and electrical signals (e.g., sensitivity) should be considered, rather than making a trade-off between these two. Third, fully functional flexible electronics that consist of different sensors in a limited space may increase crosstalk, minimizing the dimensions of the sensing elements, and integrating them with functional components such as transistors may address the aforementioned issues without weakening the signals’ readout. Finally, the slower development of rigid-substrate power and digital communication modules has created poor compatibility with flexible electronics, reflected by difficulties in the seamless integration of multiple advanced functional electronic components.

Throughout this review, we have presented the working mechanisms of humidity sensors and strain/pressure sensors, and the popular materials, structures, and fabrication methods of the sensors. Moreover, some representative examples of the sensors that respond to HMI, physical, and biological signals were described, possibly inspiring researchers to continue to develop innovations in advanced sensing materials, superior structures, and better fabrication processes. From the perspective of future developments, flexible sensors need to be highly reliable, stable, robust, accurate, and durable, as well as involving simple and low-cost manufacturing processes. Future achievements will bring us closer to an electronic era in which our perceptions can be extended with limitless possibilities.

## Figures and Tables

**Table 1:** Recent studies on humidity sensors.

Sensitive Material Type	Sensitive Material	Measurement Parameters	Production Method	Ref.
	GO	Capacitance	Screen printing	[47]
Carbon-based	CGO	Resistance	Electrospinning	[48]
	GO	Capacitance	Microwave plasma-enhanced chemical vapor deposition	[49]
	SnO_2_/RGO	Capacitance	Electrospinning	[50]
Metallic oxide or sulfide	TiO_2_	Current/voltage	Anodizing	[51]
	MoS_2_/PVP	Impedance	Inkjet printing	[52]
	RGO/WS_2_	Frequency	Sputtering	[53]
	SPEEK	Capacitance/resistance	Electrospinning	[54]
Polymer	PANI/PVDF	Impedance	Heterogeneous in-situ polymerization	[55]
	P(VDF-T rFE)/GF	Capacitance	Screen printing and spin coating	[56]

## Data Availability

Not applicable.
